# FORTA(Fit-fOR-The-Aged)-based medication optimization: retrospective analysis of experiences from an unconventional outpatient service

**DOI:** 10.1007/s41999-020-00378-z

**Published:** 2020-08-09

**Authors:** Sophia Rieg, Martin Wehling

**Affiliations:** Clinical Pharmacology Mannheim, Faculty of Medicine Mannheim, University of Heidelberg, Theodor-Kutzer-Ufer 1-3, 68167 Mannheim, Germany

**Keywords:** Fit fOR the aged (FORTA), Older people, Inappropriate prescribing, Medication optimization, Drug therapy, Outpatient service

## Abstract

**Purpose:**

To address potentially harmful polypharmacy in multimorbid older patients. Secondary to assess FORTA (Fit-fOR-The-Aged) utility in ambulatory patients.

**Methods:**

Consecutive patients attending a novel ambulatory service for gerontopharmacology were included in this retrospective analysis. Baseline data, diagnoses, previous drug treatment and recommended changes in medication were analysed and the change of the FORTA score (sum of over- and undertreatment errors) calculated.

**Results:**

The analysis of 182 cases revealed a high number of recommended medication alterations per case (9.81; 95% CI 9.13–10.50). The FORTA score was significantly reduced from 4.24 ± 2.30 to 0.80 ± 1.08 (*P* < 0.00001), as were under- and overtreatment errors if analysed separately.

**Conclusions:**

The large number of recommendations given in this special outpatient service points to a profound unmet medical need for improving medications in older patients. The FORTA approach was found to be useful to reduce both over- and undertreatment in multimorbid older patients.

## Key summary points

**Aim:**

To map prescription considerations concerning older community dwelling subjects emerging in a special gerontopharmacology hospital-based consultation.

**Findings:**

Almost 10 recommendations had to be issued per patient: drugs to be stopped, added or replaced, dosing, frequency and time of intake were optimized. Applying the Fit-fOR-The-Aged (FORTA) tool resulted in a significant improvement of under- and overtreatment errors, and the sum of errors, the FORTA score.

**Message:**

A medication review service aided by the FORTA tool detects a large number of medication errors in older outpatients and should be established in other places as well.

## Introduction

Older people represent a rapidly growing population due to demographic changes [1]. A high prevalence of multimorbidity and related polypharmacy within this group of patients poses therapeutical challenges for the physicians [2]. Additionally, age-related features of pharmacokinetics, pharmacodynamics, drug tolerance and adherence have to be addressed to ensure adequate pharmacotherapy [3].

The FORTA (Fit fOR The Aged) List and two subsequent updates have been developed in Delphi consensus procedures [5] by experts from Germany, Austria and Switzerland as a supporting tool for medication optimization in older patients. The list was clinically validated in a randomized, controlled, prospective trial (VALFORTA) which demonstrated improved quality of drug treatment and clinical endpoints, such as adverse drug effects and activities of daily living (ADL) [7].

It is the first drug list combining both negative and positive labels of drugs regarding their adequacy for older patients. After having evaluated their safety, efficacy and age appropriateness, the drugs were classified into four categories from A (indispensable) to B (beneficial), C (questionable) and D (avoid). The current list contains 296 assessments for 29 indications [6]. The sum of medication errors by counting missing, but necessary drugs (undertreatment), unnecessary drugs (overtreatment) or drugs with suboptimal FORTA labels (mistreatment) is indicated by the FORTA score [7]. It can, therefore, be used to numerically measure the quality of prescription and the impact of the related interventions. The application of FORTA requires in-depth knowledge of the patients’ medical history, relevant diagnoses, functional condition, wishes and former drug treatments [8]. So far, the FORTA approach has been tested only in a clinical setting.

The aim of the present paper is to describe an outpatient service for gerontopharmacology that is unique to Germany and to report on the first experiences regarding its impact on prescription quality in older outpatients; particular emphasis lies on the FORTA tool and its impact on medication list optimization in this patient group.

## Methods

### Description of the outpatient service for gerontopharmacology

The first outpatient service for gerontopharmacology in Germany was established at the Center of Geriatric Medicine in Mannheim in 2008. This center was founded in 2000 as the first of its kind in Germany by the head of the geriatric clinic and the senior author (MW). MW who is a board-certified internist, cardiologist and clinical pharmacologist, is the physician in charge; secretarial assistance was provided by two personal assistants, nurses were not involved. Supervision and critical upraisal were provided by geriatricians from the clinics for geriatrics. To be admitted, patients seeking advice regarding their medication have to be 65 + years old and to take five drugs or more regularly. Patients were instructed to bring all relevant medical records, updated medication plans and—if possible—a referral by their general practitioner (GP). Most patients are self-referrals, less than 10% were referred by the GP.

A typical consultation takes about 1–1 1/2 h; in about 80% of cases, relatives accompany the patients and support the history taking and description of the current health status and complaints.

### Inclusion criteria and participant flow

The inclusion criteria for this analysis and those for admission to the service were identical (see above): patients have to be 65 + years old and to take five drugs or more regularly. Yet, due to inconsistencies in admission control, few patients were seen at the service that did not meet its admission criteria. They were excluded from this analysis.

A total of 199 consecutive patients seen by the senior author from 2008 until 2020 were checked for inclusion, 9 patients were seen twice.

After the exclusion of patients younger than 65 (15 cases, 7.4%), patients taking less than 5 medications regularly (5 cases, 2.5%) and of cases with incomplete documentation (two cases, 1.0%), 182 visits (89.2%) by 173 patients (80 male, 93 female) were analyzed and counted as 182 cases. The second consultation was considered as a new case as it occurred in average 2.56 years after the first one. Of course, observations for the 2nd visit were not independent of those obtained at the first visit, but omitting the 2nd visit did not qualitatively change any conclusion as checked for key results.

### Data acquisition and analysis

Information about the patients’ medical history, preliminary findings and previous therapy were collected from the available documents, the physical examination and the patients’ interviews and—in the most cases—the accompanying relatives. A particular emphasis was laid on the assessment of current medications. Former medications were recorded as well to gain information on failed or intolerable therapeutic approaches. Formal, standardized medication schemes detailing drug names, doses, intervals and related diagnoses were presented in the most cases, or were newly devised if missing. Critical upraisal of current medication schemes resulted in corrections for all cases.

For this retrospective analysis, a database was generated after exclusion of patients younger than 65 years or regularly taking less than five drugs and those without proper documentation. It contains the patient’s baseline characteristics (both anthropometric and biochemical data), medical history (especially therapeutically and prognostically relevant diagnoses) and previous drug therapy. Furthermore, medication recommendations concerning deprescribing, addition or replacement of drugs, changes in dose, timing and frequency of intake and type of application were collected. Recommendations were further subdivided in direct/non-conditional advices and indirect/conditional advices. These drug-related recommendations were excerpted from the final doctor’s letter sent to the physician providing continuous care (in most cases a GP) and to the patient 2–8 days after the service. Changes in the drug therapy of the most recent 46 patients seen from January 2016 to June 2019 were analysed by the determination of the FORTA score (as described above). The FORTA 2015 list [4] was used, and the FORTA score before and after the consultation was generated.

The restriction of the patient sample for the FORTA analysis is due to the fact that a FORTA list became only available in 2014 though the service started in 2008. In 2016 the 2nd, largely ameliorated version (FORTA2015) was published, and we decided to analyse only patients from January 2016 on for the following reasons: few patients that could have been added from 2014 would have increased heterogeneity due to changed FORTA entries. Applying the FORTA-list to even earlier patients (from the beginning of the service in 2008) would be worse, as the coverage of the list and the validity of the FORTA labels would not have matched with drug use in older patients in those years, thereby further increasing heterogeneity. For this reason, the FORTA list has to be actualised frequently and its most recent edition should be used to adequately reflect current assessments. Besides, FORTA-independent recommendations (e.g. changes in dose, timing, route of application, or those drugs/diagnoses not covered by FORTA) were collected for all patients.

The analysis was approved by the ethics committee at the Medical Faculty Mannheim, Heidelberg University (2018-887R-MA).

### Statistical methods

All data are expressed as mean ± standard deviation or 95% confidence interval (CI) as indicated. Comparisons were tested by the Wilcoxon test; statistical significance was assumed when *P* < 0.05. Correlations were calculated by linear regression analysis using Microsoft Excel.

## Results

### Baseline data

The mean patient age was 76.76 ± 6.18 years (median 76). The number of therapeutically or prognostically relevant diagnoses was 6.19 ± 2.18 (95% CI 5.87–6.50) and the number of drugs was 10.97 ± 3.73 (95% CI 10.43–11.52). Initially, 40% of the cases had a medication count of five or more prescribed drugs—defined as polypharmacy—and 60% took ten drugs or more—defined as severe polypharmacy.

Baseline characteristics and laboratory values of all cases are presented in Table 1.

**Table 1:** Baseline characteristics

Characteristic	Mean ± SD	Minimum	Maximum
Age [years]	76.76 ± 6.18 (*n* = 182)	65	91
Male [number/%]	80/46.2		
Female [number/%]	93/53.8		
Number of diagnoses	9.87 ± 3.07	3	21
Whereof prognostically/therapeutically relevant	6.19 ± 2.18 (*n* = 182)	1	14
Number of drugs	10,97 ± 3.73 (*n* = 182)	5	27
Weight [kg]	80.11 ± 15.52 (*n* = 125)	48	117
Height [cm]	167.10 ± 8.87 (*n* = 116)	148	187
BMI [kg/m^2^]	28.69 ± 4.48 (*n* = 116)	20.00	40.40
Hospitalization in the past 3 years [number]	1.65 ± 0.88 (*n* = 97)	1	4
SBP [mmHg]	137.02 ± 22.62 (*n* = 177)	70	220
DBP [mmHg]	77.37 ± 12.77 (*n* = 173)	50	120
HR [beats/min]	70.09 ± 10.31 (*n* = 144)	48	110
Creatinine clearance [ml/min]	60.88 ± 22.38 (*n* = 140)	14.00	126.96
Total cholesterol [mg/dl]	195.01 ± 58.53 (*n* = 67)	77	329
LDL-cholesterol [mg/dl]	119.95 ± 44.59 (*n* = 129)	26	300
Plasma potassium [mmol/l]	4.38 ± 0.55 (*n* = 138)	2.92	7.40
HbA1c [%]	6.68 ± 1.21 (*n* = 99)	4.70	11.30

*BMI* body mass index, *SBP* systolic blood pressure, *DBP* diastolic blood pressure, *HR* heart rate, *LDL* low dense lipoprotein

Body mass index was above 30 kg/m^2^ in 29.3% of the cases. Systolic blood pressure was 140 mmHg and higher in 51.4% of the cases and diastolic blood pressure 60 mmHg and lower in 15.0%, respectively. Mean creatinine clearance was 60.88 ± 22.38 ml/min (range 14.00–126.96) and was below 30 ml/min in 6.4%. LDL cholesterol was above 160 mg/dl in 17.8% und HbA1c above 8% in 13.1%. Hypokalaemia (< 3 mmol/l) was present in 0.7%.

### Drug therapy

**Fig. 1:**
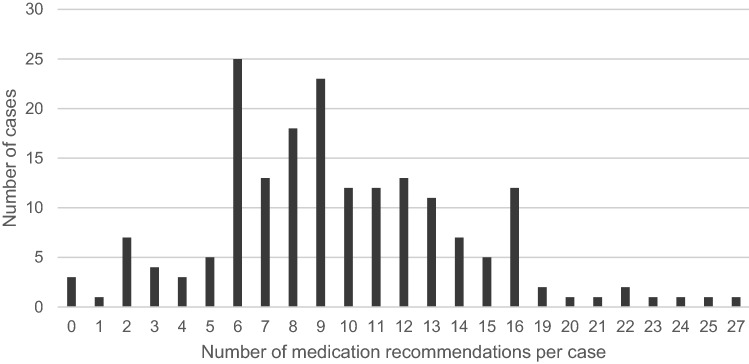
Medication recommendations per case. Data shown as number of cases (*n* = 182) with corresponding number of given recommendations

The mean number of recommendations per case was 9.81 ± 4.74 (95% CI 9.13–10.50). These include prescription of drugs in case of undertreatment, deprescription of drugs in case of overtreatment, replacement by more appropriate drugs, changes in dosing and recommendations concerning time and frequency of intake. 50% of the cases received between six and ten medication recommendations (Fig. 1). The numbers of individual change recommendations show an impressively wide range from 0 (3 cases) to 27 (1 case).

A strong correlation between the initial number of drugs and the number of medication recommendations was found (*P* < 0.0001, regression equation $y=0.5232x+4.1058$).

**Fig. 2:**
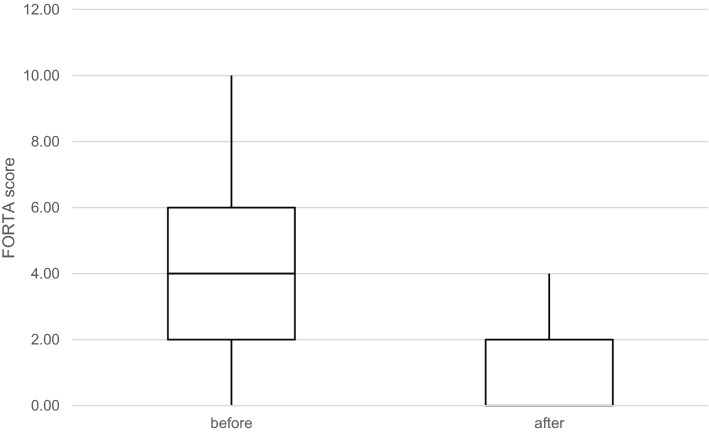
FORTA score before and after medication optimization in a box-whisker-plot. Calculation base are data of the most recent 46 patients.* P* < 0.00001, Wilcoxon-test.

The FORTA score (sum of errors classified as under-, over- and mistreatment per patient) changed from 4.24 ± 2.30 to 0.80 ± 1.08 (significant at *P* < 0.0001, Fig. 2). There was a significant decrease (*P* < 0.0001) for both undertreatment (from 2.15 ± 1.37 to 0.33 ± 0.51) and overtreatment (from 2.26 ± 1.51 to 0.48 ± 0.62). FORTA A drugs significantly increased from 3.59 ± 1.97 to 4.37 ± 2.09 (*P* < 0.001) and FORTA D drugs significantly decreased from 0.57 ± 0.90 to 0.09 ± 0.28 (*P* < 0.001).

**Fig. 3:**
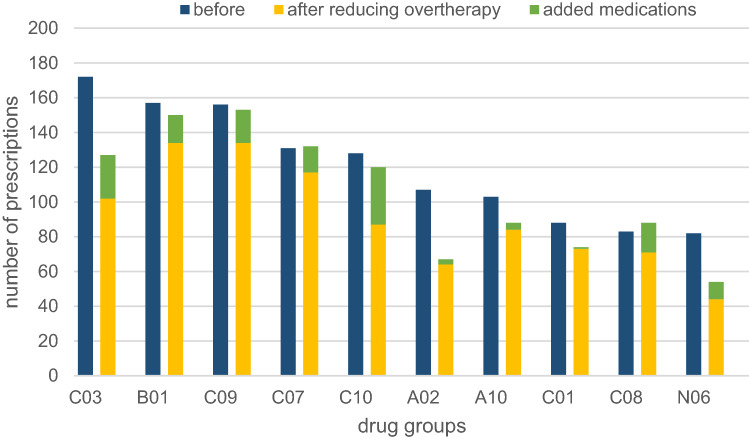
Frequency of prescribed drug groups at the ATC system level before and after medication evaluation. Data shown as numbers of prescriptions per group (*n* = 182). Numbers before evaluation are given in blue columns; after evaluation, they are further subdivided into unchanged medications without those that had to be removed (yellow columns) and added medications (green columns). *ATC system* anatomical-therapeutical-chemical system, *C03* diuretics, *B01* antithrombotic agents, *C09* agents acting on the renin-angiotensin-system, *C07* beta-blocking agents, *C10* lipid-modifying agents, *A02* drugs for acid related problems, *A10* drugs used in diabetes, *C01* cardiac therapy, *N06* psychoanaleptics, *C08* calcium channel blockers

Changes of drugs at the anatomical-therapeutical-chemical (ATC) system level are shown in Fig. 3. Initially, diuretics (C03) were the most common drugs (172 prescriptions, 8.6% of prescribed drugs). In the course of medication optimization, diuretics stop was recommended in 70 of the cases (amongst them hydrochlorothiazide 31 times); a prescription of diuretics was proposed in 25 cases so that diuretics were finally recommended in 127 cases (7.3% of prescribed drugs).

Another example for significant reduction relates to proton pump inhibitors (A02): initially, there were 107 prescriptions, which were reduced by 37% after evaluation (67 cases remaining).

For lipid-modifying agents (C10) no prominent difference in the total number of prescriptions prior to and after therapy evaluation was found. However, Fig. 3 shows that 41 medications were stopped (among them simvastatin 17 times and ezetimibe 10 times), but in 33 cases the C10-substances (particularly atorvastatin 14 times and simvastatin 7 times) were recommended to be added in the therapy which could explain the insignificant change of the total number of C10-drugs.

Table 2 depicts the numbers of direct and conditional (e.g. depending on the clinical course or tolerability) recommendations, splitted into addition or stop of drugs, changes in doses or other modalities (frequency, interval, route of application). Obviously, twice as many drugs were directly recommended to be stopped as compared to those to be started.

**Table 2:** Number of recommendations

	Addition	Deletion	Dosing	Others (frequency, timing of intake, route of application)	Total
Direct recommendations (Mean ± SD)	1.46 ± 1.32	2.81 ± 2.39	0.82 ± 0.92	0.38 ± 0.77	5.42 ± 3.21
Conditional recommendations (Mean ± SD)	1.93 ± 1.43	1.31 ± 1.24	1.20 ± 1.29		4.39 ± 2.66
					9.81 ± 4.74

*SD *standard deviation

For the 46 most recent patients 3.61 ± 2.38 recommendations on the choice of drugs were based on the FORTA list, 2.35 ± 1.48 recommendations concerning drugs and/or diagnoses not covered by the FORTA list. The conditional recommendations were not collected in this analysis which could explain the lower number of therapy advices in total (5.96 ± 2.75) as compared to the number of recommendations given for the entire database.

## Discussion

Suboptimal and potentially dangerous medication schemes in older patients represent a serious problem in older patient care. They have been addressed by various strategies including drug listing approaches which have gained considerable interest. In a recent review, we detected 73 different listing approaches and grouped them into those starting at the patient’s level (patient-in-focus-listing approaches, PILA) and those focussing on the drug list itself (drug-oriented listing approaches DOLA) [9]. The clinical validation of PILA was superior to DOLA in that most trials on clinical endpoints were successful, whereas those on DOLA failed to be validated.

FORTA is one of the successfully validated PILA, as it is also the case for START/STOPP [10]. In both cases, the validation studies were done in hospitalized patients.

In comparison, little is known about the utility of such approaches in the outpatient setting/ambulatory care. The negative impact of potentially inappropriate medications has been robustly demonstrated in older outpatients as well [11]. The main challenge, however, remains how to address this problem. In a recent review [12] 7 interventional studies were included addressing the clinical improvement of primary care patients by medication optimization. The outcome was disappointing: it found no statistically significant benefit from any interventions on e.g. hospitalisation, mortality, mental health and other clinical endpoints. Of course, discharge medications are important for primary care and GPs often adhere to hospital-based recommendations [13].

A potential approach for better outcomes could be an outpatient service dedicated to gerontopharmacology; this did not seem to exist in Germany until 2008, and still most likely does not exist elsewhere. At least, a Medline search on these key terms did not show any related entry through this search may not have been sensitive enough. In 2008 the senior author established a service directly addressing the issues of polypharmacy in older outpatients. So far, this service is unique for Germany, and upon 10 years after its invention the impact on medication in older patients should be evaluated by this analysis. Obviously, it is a one-time medication review service, and the real impact on the long-term medication of attending patients cannot be investigated. Thus, the analysis of medication recommendations reported here could only be seen and evaluated under the assumption that they are performed in the ongoing treatment plans for the patients.

From this point of view, these findings shed an impressive light into the main issue: drug treatment was found to be inappropriate to an extent that exceeds expectations. Almost 10 recommendations on average were given to address a single patient’s problems. As a matter of fact, almost half of them (45%) were secondary recommendations (‘in case of’) and it still remains unclear whether they were needed or redundant.

With the increasing appreciation and refinement of the FORTA approach, its use as an assessment tool demonstrates that it may be used to guide and assess medication optimization (FORTA score as scale) in outpatients as well.

The results reported here raise severe issues in medication management for older patients. To our knowledge, a utility for an outpatient service which could address the above mentioned problems has not been acquired by any other ambulatory setting in Germany, so far.

From those experiences reported here, the senior author tentatively identifies two main reasons for this lack of appreciation though they are still speculative:

1. Remuneration: the service generates revenues of around 20 EUR per case, but requires 1 1/2—2 h of a highly skilled and trained MD (who works for free). Any doctor who has to make a living from this service would be bankrupt in a few weeks time.
2. Some GP do not accept such service/tool/utility as can be seen in the low referral numbers by GP. The reason is speculative, but the service could be interpreted as a threat which would possibly unveil insufficiencies of medical care. The question is: how could a GP with a reimbursed consultation time of 7 min improve their medication management? That should be rather seen as a healthcare system deficit, which the GPs should not be blamed for.

Noteworthy, more prospective studies to assess the applicability of PILA such as FORTA or START/STOPP in the ambulatory setting will be needed. A major limitation for conducting such studies is the acquisition of suitable funding.

### Limitations

The sample of patients was biased by the criteria of access to the service. Therefore, a number of diagnoses and drugs, frequency of drug intolerance are probably higher as compared to the average of older patients.

This retrospective study only includes a sample of 182 cases in total, medication management was evaluated by using the FORTA score for only 46 cases. Furthermore, a control group and randomization were not assigned to the study, as well. Yet, the service reduced the total FORTA score and its over- and undertreatment components at a *P* < 0.0001. Even changes of FORTA A and D drugs were significant at a *P* < 0.001. We observed this highly significant effect of a FORTA intervention in the randomized controlled VALFORTA trial as well [7].

Data concerning the patients’ medical history, existing diagnoses and previous drug therapy was only extracted from medical reports provided by the patient, no clinical diagnostics happened during this service.

The present analysis of recommendations would lead to real improvements in the medication plans only if enacted by the GP; such data have not been collected.

Personal bias by the senior author is another limitation of the study.

## Conclusion

The study clearly demonstrates the need for optimizing drug treatment of the aged and thereby endorses the potential of applying the FORTA tool. Although the recommendations from this unique service have been formally validated by the FORTA score, their practical implementation still remains unknown. Yet, such services appear to be useful at a larger scale and should be implemented in the regular outpatient/inpatient services elsewhere as well.
